# Nanozinc Ecotoxicity in the Freshwater Invasive Bivalve *Limnoperna fortunei* Under a Climate Change Scenario

**DOI:** 10.3390/ani15182734

**Published:** 2025-09-19

**Authors:** Analía Ale, Victoria S. Andrade, Florencia M. Rojas Molina, Luciana Montalto, Lucía M. Odetti, Pablo E. Antezana, Martín F. Desimone, María Fernanda Simoniello

**Affiliations:** 1 Cátedra de Toxicología, Farmacología y Bioquímica Legal, Facultad de Bioquímica y Ciencias Biológicas, Universidad Nacional del Litoral (FBCB-UNL), Santa Fe S3000, Argentina; luodetti@gmail.com (L.M.O.); fersimoniello@yahoo.com.ar (M.F.S.); 2 Consejo Nacional de Investigaciones Científicas y Técnicas (CONICET), Buenos Aires C142F, Argentina; 3 Instituto Nacional de Limnología, Consejo Nacional de Investigaciones Científicas y Técnicas (CONICET), Universidad Nacional del Litoral (INALI-CONICET-UNL), Santa Fe S3000, Argentina; vandrade@inali.unl.edu.ar (V.S.A.); florojasm@yahoo.com.ar (F.M.R.M.); lmontalto@inali.unl.edu.ar (L.M.); 4 Facultad de Humanidades y Ciencias, Universidad Nacional del Litoral (FHUC-UNL), Santa Fe S3000, Argentina; 5 Instituto de Bioquímica y Medicina Molecular, Facultad de Farmacia y Bioquímica, Universidad de Buenos Aires (FFyB-UBA), Consejo Nacional de Investigaciones Científicas y Técnicas (CONICET), Buenos Aires C1113, Argentina; pablo.e.antezana@gmail.com; 6 Instituto de Química y Metabolismo del Fármaco, Facultad de Farmacia y Bioquímica, Universidad de Buenos Aires (IQUIMEFA-FFyB-UBA), Consejo Nacional de Investigaciones Científicas y Técnicas (CONICET), Buenos Aires C1113, Argentina; desimone@ffyb.uba.ar

**Keywords:** filtration rate, golden mussel, oxidative stress, temperature, tissue damage

## Abstract

In a changing world where temperature is expected to increase, emerging nanopollutants could affect the biota in complex ways. With zinc oxide nanoparticles (ZnONP) being one of the most applied nanomaterials, we exposed the freshwater invasive bivalve *Limnoperna fortunei* to 0 (control), 25, and 250 µg/L of ZnONP at 27 or 31 °C for 96 h. In parallel, a 24 h bioassay was performed to calculate filtration rate. After 96 h, in soft tissue of the bivalves, tissue-damage-related enzyme activities (aspartate aminotransferase and alkaline phosphatase) were inhibited at both concentrations and temperatures. Oxidative stress was observed through increased superoxide dismutase activity after both ZnONP concentrations at 27 °C and decreased catalase activity after 250 µg/L at 31 °C, while glutathione-S-transferase activity showed opposing significant tendencies depending on temperature. After 6 h, the filtration rate differed significantly between control groups, as it was higher at 31 °C. However, in case of 31 °C, bivalves exposed to ZnONP drastically decreased their filtration rate compared to control. Our study highlights nanotoxicological implications of ZnONP; as even at environmentally relevant concentrations (such as the lowest applied in this study), they exert deleterious effects on freshwater organisms, which could be worsened in a climate-change scenario.

## Simple Summary

Zinc oxide nanoparticles (ZnONP) are among most applied nanomaterials worldwide. When released into aquatic environments, they exert ecotoxicological effects on associated biota. Climate change affects every corner of the world, with temperatures expected to rise by up to 4 °C by the end of this century. Therefore, we aimed to evaluate a battery of biomarkers in soft tissue (enzyme tissue damage and oxidative stress) and filtration rate in the freshwater invasive bivalve *Limnoperna fortunei* after exposure to 0 (control), 25, and 250 µg ZnONP/L at 27 and 31 °C. Inhibition of tissue-damage-related enzyme activities was evidenced at both temperatures. Significant changes were observed in oxidative-stress-related enzyme activities, which depended on the temperature evaluated, while no lipid peroxidation was evidenced. Filtration rate showed differences between control groups at both temperatures, and a significant decrease was observed at 31 °C after exposure to both ZnONP concentrations.

## 1. Introduction

Metal-based nanomaterials (NM), including nanoparticles (NP), have been extensively studied and applied in various sectors due to their unique properties. In particular, zinc oxide nanoparticles (ZnONP), or nanozinc, are third most produced NM after silica and Ti-based ones, with a global production of up to 36,000 tons per year [1]. Nanozinc has diverse applications and is employed, for example, in solar cells, optoelectronic devices, biomedicine, antibacterial materials, and personal care products [2]. In parallel with their growing production and applications, it has been estimated that the most important ZnONP flow (95%) goes to wastewater (mainly through prevalent use in cosmetics) and ultimately reaches aquatic environments [3,4]. However, predicted environmental concentrations for surface water are in the low range of ≤0.17 µg/L for European rivers [5], or 0.5 µg/L for the European Union according to the NanoFATE project, while in municipal wastewaters these values could reach 10 µg/L [6].

As with many of the NM studied in nanotoxicity research, when ZnONP become available to non-target biota they may exert deleterious effects and alter their natural homeostasis [4,7,8]. In this sense, benthic invertebrates, especially filter and suspension feeders, have gained importance in nanotoxicology because when they reach the natural aquatic systems, NM are likely to form agglomerates which become deposited and accumulated in sediment, increasing the biological risk for the associated organisms [4,9]. Nevertheless, nanotoxicity in benthic organisms has been poorly explored, with emphasis on freshwater systems [4,10]. Moreover, in comparison with other NP, nanozinc has been insufficiently addressed as reviewed by Cazenave et al. [11] and Gutierrez et al. [9], who explained that only 11% and 12% (approximately) of studies conducted on fish and invertebrates, respectively, evaluated ZnONP toxicity among other metal-based NP (with Ag- and TiO_2_NP being the most evaluated ones). Few studies on freshwater mollusks have revealed deleterious effects after nanozinc exposure. It has been reported that different ZnONP concentrations generated morphological changes, bioaccumulation, inflammation, genotoxicity, and neurotoxicity in *Dreissena bugensis* and *Limnoperna fortunei* [12,13]. Filtration rate under exposure to NP has been poorly addressed, despite the fact that it constitutes a key tool for evaluating toxicity. In this sense, a decrease in such behavior was evidenced in the case of high concentrations of ZnONP (2.5–25 mg/L) in the marine mussel *Mytilaster lineatus* and the estuarine one *Dreissena polymorpha* [14].

In the context of increasing emerging nanopollutants, climate change reaches every corner of the Earth with its imminent threats to the natural systems, particularly the freshwater ones [15,16,17]. The Intergovernmental Panel on Climate Change (IPCC) [18] reported an increase in the mean atmospheric temperature up to 4 °C by the year 2100. In this sense, a recent review carried out by Gutierrez et al. [19] explains that temperature increase within the framework of climate change is remarkably associated with synergistic toxic interactions with other pollutants and stressors, and emphasizes the need for intensifying research or emergent contaminants in a changing world.

*Limnoperna fortunei*, commonly named the “golden mussel”, is a freshwater invasive bivalve species established and naturalized in the aquatic ecosystems worldwide [20]. Therefore, due to its widespread distribution, high availability, size, and sedentary lifestyle, this species has been used as test organism for laboratory studies to evaluate the toxicity of many kinds of pollutants, including NP [13,21,22,23]. The aim of this study was to assess the ecotoxicity of the emerging nanopollutant ZnONP (0, 25, and 250 µg/L) in the golden mussel (*L. fortunei*) under different temperatures in a climate change context (27 and 31 °C). For this purpose, a battery of biomarkers was evaluated, which consisted in biochemical parameters (enzyme tissue damage and oxidative stress) and behavioral responses (filtration rate). The novelty of this work lies in addressing the lack of ecotoxicological assessments on freshwater mollusks, despite their known sensitivity to nanopollutants, under a scenario representing a warming world.

## 2. Materials and Methods

### 2.1. Nanozinc Characterization

The nanoparticles were purchased from Sigma-Aldrich^®^ (Steinheim, Germany) (Product number 721077), guaranteeing their purity and stability. According to the Certificate of Analysis, the particles were correctly dispersed in the medium (H_2_O), with a pH 8.9, and were 40 nm-sized. The stock concentration was reported to be 19% wt.

ZnONP were characterized by transmission electron microscopy (TEM) using a Zeiss EM109T (Oberkochen, Germany) electron microscope. Briefly, a drop of the sample was placed on carbon-coated copper grids and allowed to dry for a few minutes. The hydrodynamic diameter and zeta potential of the NP were also determined using a Brookhaven Instruments dynamic light scattering (DLS) instrument (Nashua, NH, USA). For statistical accuracy, all measurements were performed in triplicate.

### 2.2. Bivalves and Exposure Conditions

During the summer season (March 2024), *L. fortunei* adults (mean length: 26.59 ± 0.41 mm; weight: 1.40 ± 0.04 g; N = 100) were manually collected from a dock located in the Santa Fe River (a secondary channel of the Middle Paraná River, 31°38′34.90″ S; 60°41′6.22″ W) (Figure S1). The acclimation procedure followed that described by Cazenave [22]. The organisms were transferred to the laboratory in plastic containers filled with natural river water, separated, and brushed to remove the biofilm. The animals were randomly separated into two groups, and for the first 24 h, they were kept in a medium composed of half natural river water and half with dechlorinated tap water, with continuous oxygenation at 27 or 31 °C (in incubators). Then, the bivalves were kept in dechlorinated tap water for 48 h, under a 16/8 h photoperiod (light/darkness) and with algae-based food (*Tetradesmus obliquus* algae culture) ad libitum until 24 h before starting the final experiments. When an individual was attached and showed signs of valve activity in response to physical stimuli, it was considered healthy. The temperatures were chosen according to the mean temperature in their habitat during the summer season, the optimal temperature reported for the species, and the tolerance limit for survival [24,25,26], plus 4 °C according to the IPCC predictions [18]: 27 and 31 °C. The tap water conditions were monitored during the entire acclimation period and experiments, which remained consistent (pH 7.1 ± 0.03, conductivity 261.25 ± 16.02 µS/cm, constant aeration), by employing a portable multiparameter device (Hach^®^ HQ4300) (Loveland, CO, USA).

The ZnONP concentrations were selected according to the LC50-96 h value estimated as 11.31 mg/L (confidence interval: 5.39–17.23) (Figure S2) [27], representing ~2% and ~0.2%. For this purpose, four nanozinc concentrations were selected (plus a control group) with a dilution factor of 10: 0.025, 0.250, 2.50, and 25 mg ZnONP/L. Furthermore, the lowest concentration was considered to be environmentally relevant as the predicted Zn concentration for urban surface waters is on the order of 10–50 µg/L [28,29].

### 2.3. Experimental Designs

One battery of bioassays consisted of 96 h exposures to 0 (control), 25, and 250 µg ZnONP/L at 27 or 31 °C. Each experimental unit was composed of 5 individuals (in 250 mL aquaria with dechlorinated tap water and constant aeration) and was replicated five times. Renewals of all treatments were made every 24 h. The exposure was conducted by directly adding ZnONP into the aquaria after preparing the corresponding dissolutions (in ultrapure water), which were prepared daily prior to their application to prevent any particle transformation and/or agglomeration. The animals were not fed during the experiment, and the photoperiod was consistent with the acclimation process (16/8 h light/darkness). At the end, the soft tissue of each animal was removed and stored at −80 °C until further analysis.

Separately, a second battery of bioassays aimed to calculate the filtration rate of *L. fortunei*. It consisted of 24 h exposure to the aforementioned concentrations and temperature conditions in the absence of light (to prevent algal growth). Each experimental unit consisted of a 250 mL aquarium with a predetermined algae concentration (*T. obliquus*) and two individuals, which was replicated five times for each ZnONP concentration and temperature. Phytoplankton samples were taken from each aquarium at 0 (at the beginning before the bivalves’ addition), 3, 6, 12, and 24 h (end of experiment) and fixed with a 1% acidified Lugol’s solution. The number of algae was counted in a Neubauer chamber under a compound microscope. Furthermore, experimental units containing only algae (without bivalves) were included as controls to assess algal growth in the aquaria, since their data were used to correct initial algal concentrations in the filtration algorithm. Before starting this experiment, a flocculation test (with algae and ZnONP) was conducted to verify that the ZnONP did not alter the algae dispersion or behavior in the medium [30].

### 2.4. Tissue Damage and Oxidative Stress-Related Biomarkers

For the 96 h exposure, the soft tissue of each bivalve was homogenized to determine enzyme tissue damage and oxidative stress markers (n = 5 per biomarker type) [31,32]. Enzyme activities of alanine aminotransferase (ALT), aspartate aminotransferase (AST), and alkaline phosphatase (ALP) were measured using the methodology proposed by Reitman & Frankel [33] with commercial kits (Wiener Lab^®^) (Rosario, Argentina). Oxidative stress was assessed by determining the enzyme activities of superoxide dismutase (SOD) [34], catalase (CAT) [35,36], and glutathione S-transferase (GST) [37]. Lipid peroxidation levels (LPO) were determined using the thiobarbituric acid reactive substances (TBARS) assay according to Yagi [38]. Each sample was measured in triplicate, and all results were expressed relative to protein content determined with a commercial kit (Wiener Lab^®^).

### 2.5. Filtration Rate

The filtration rate of *L. fortunei* was estimated based on the predetermined algae concentration within the treatments at the following time points: 3, 6, 12, and 24 h for each temperature (27 or 31 °C), following the equation proposed by Jørgensen [39]:

$$F=V\cdot \;\;[\frac{\ln\left(\frac{Ci}{Cf}\right)-\;ln(\frac{C^{\prime }i}{C^{\prime }f})}{N\;\cdot \;\;T}]$$

where *F* is the filtration rate (mL/individual/h), *V* is the volume of water in the experiment (mL), *N* is the number of organisms per aquarium, *T* is the total filtration time (h), *C_i_* and *C_f_* are the algae concentrations with bivalves at the beginning and end of each time period, and *C′_i_* and *C′_f_* are the algae concentrations in the control aquarium (without bivalves) for the beginning and end of each time period.

### 2.6. Statistical Analysis

The lethal concentration 50 (LC50) was estimated based on the lethality data recorded after 96 h of ZnONP exposure through Probit analyses performed with the “drc” R studio package (version 2025.05.0+496) [40].

Data are reported as mean ± standard error. Shapiro-Wilks and Levene’s tests were applied to evaluate normality and homogeneity of variance, respectively. Variables without normal distribution were transformed using log10 and tested again, prior to parametric analysis. For statistical comparisons among the treatments at each temperature, 1-way ANOVA was performed, considering a significance of *p* < 0.05. Then, Tukey post-test was used for normally distributed data, and the Kruskal–Wallis test for non-normally distributed data. To evaluate interactions between treatments and temperature, a 2-way ANOVA (*p* < 0.05 significance) was employed. In these cases, statistical analysis was performed using the InfoStat software (version 2008) (Universidad Nacional de Córdoba, Argentina).

The filtration rate data were analyzed by applying a generalized linear mixed model (GLM) with repeated measures, lognormal distribution, and Tukey post-test with glmmTMB package in R Studio software (version 2025.05.0+496).

## 3. Results

### 3.1. Nanozinc Characterization

**Figure 1:**
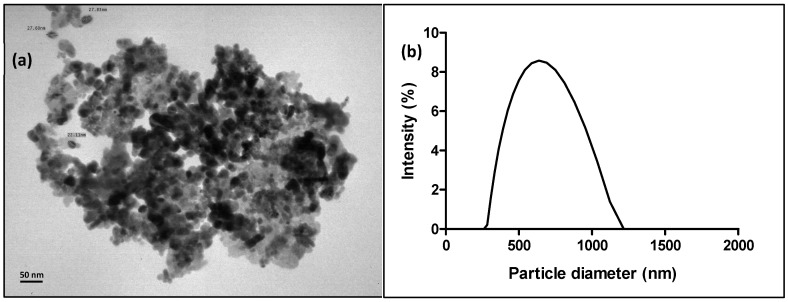
(**a**) TEM and (**b**) DLS analyses of the ZnONP.

Transmission electron microscopy (TEM) revealed that the ZnONP had an average primary diameter of approximately 27 nm. Despite this small size, a noticeable degree of agglomeration was present in the images (Figure 1a). Further analysis by dynamic light scattering (DLS) confirmed the extensive aggregation of the nanoparticles in aqueous suspension. The measured hydrodynamic diameter was significantly larger, at 630 ± 207 nm, highlighting the formation of aggregates in the colloidal system (Figure 1b). To assess the stability of these aggregates, we measured the zeta potential, which was found to be −26.09 ± 0.79 mV. This value falls within the range of moderate colloidal stability, meaning the electrostatic repulsion is insufficient to prevent aggregation over time, particularly with changes in pH or ionic strength [41,42]. The combined DLS and zeta potential data therefore indicate that the nanoparticles are prone to dynamic aggregation in solution, which can significantly influence their bioavailability and ultimate toxicity.

### 3.2. Tissue Damage and Oxidative Stress-Related Biomarkers

**Figure 2:**
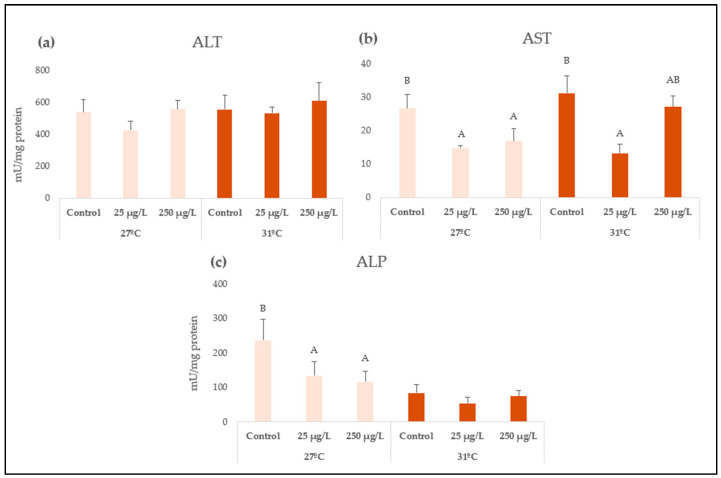
Tissue-damage-related enzyme activities: (**a**) alanine aminotransferase (ALT), (**b**) aspartate aminotransferase (AST), and (**c**) alkaline phosphatase (ALP) in soft tissue of *L. fortunei* exposed to 0, 25 and 250 µg ZnONP/L at 27 or 31 °C for 96 h. The values are expressed as means ± SE. Means not sharing the same capital letter (A or B) are significantly different at *p* < 0.05.

After 96 h of exposure to ZnONP at 27 °C, decreased enzyme activities of AST and ALP were observed after both concentrations (*p* = 0.0484 and *p* = 0.0467, respectively), while no changes were observed for ALT. At 31 °C, AST enzyme activity also significantly decreased only after the lowest nanozinc concentration (25 µg/L) (*p* = 0.0125), while no changes were observed for either ALT or ALP enzyme activities (Figure 2).

**Figure 3:**
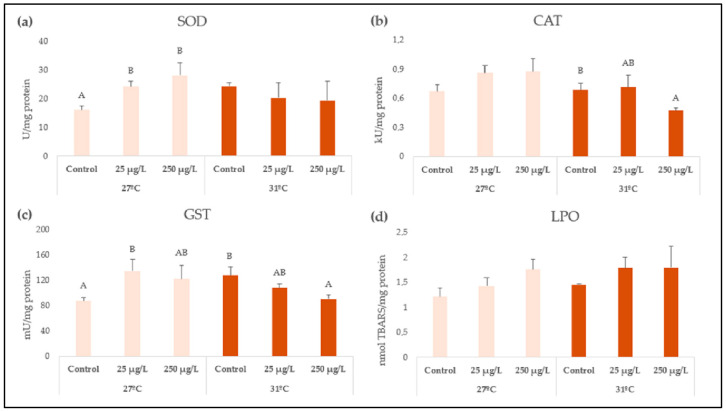
Oxidative stress-related enzyme activities: (**a**) superoxide dismutase (SOD), (**b**) catalase (CAT), and (**c**) glutathione S-transferase (GST), and (**d**) lipid peroxidation levels (LPO) in soft tissue of *L. fortunei* exposed to 0, 25 and 250 µg ZnONP/L at 27 or 31 °C for 96 h. The values are expressed as means ± SE. Means not sharing the same capital letter (A or B) are significantly different at *p* < 0.05.

Oxidative stress-related biomarkers are shown in Figure 3. At the lowest temperature, while no changes were observed for CAT, SOD enzyme activity was increased after exposures to both nanozinc concentrations (*p* = 0.0083), and GST also showed increased activity, which was significant in the case of 25 µg/L. Conversely, at the highest temperature, CAT and GST enzyme activities decreased at the highest concentration (250 µg/L) (*p* = 0.0305, *p* = 0.0487, respectively); however, SOD showed no difference among the treatments. Interactions between the temperature and nanozinc concentrations were significant for SOD (*p* = 0.0026), CAT (*p* = 0.0373), and GST (*p* = 0.0015) enzyme activities (Table 1). Finally, LPO levels showed no differences in comparison with their respective control groups.

### 3.3. Filtration Rate

**Figure 4:**
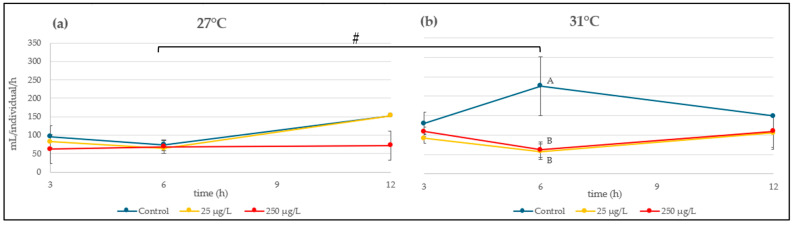
Filtration rate of *L. fortunei* exposed to 0, 25 and 250 µg ZnONP/L at (**a**) 27 or (**b**) 31 °C for 24 h. The values are expressed as means ± SE. Means not sharing the same capital letter (A or B) are significantly different at *p* < 0.05. The hash sign (#) means statistical difference between filtrations rates in the control groups at 27 or 31 °C at *p* < 0.05.

The filtration rate mean values are shown in Figure 4. Data at 24 h were excluded from the analysis as no algae were observed at this time. A significant interaction between temperature and time was observed (h) (*p* = 0.0086). After 6 h, the control filtration rate was significantly higher at 31 °C compared to 27 °C (*p* = 0.0196). Furthermore, after 6 h at the highest temperature, the filtration rates of *L. fortunei* were drastically decreased following exposure to both ZnONP concentrations (25 and 250 µg/L) in comparison to the control group (*p* = 0.0215 and *p* = 0.0198, respectively). A summary of the GLM modeling results is shown in Table 2.

## 4. Discussion

Zinc oxide nanoparticles are among the most produced NM worldwide, despite the fact that their ecotoxicology has been poorly addressed within the available literature [1,9,11]. The high volumes of release into aquatic environments have posed them as a threat to both humans and ecosystems [43]. In particular, we emphasize the need for assessing nanozinc toxicity under freshwater conditions and in benthic organisms like mussels, as they were stated to be particularly sensitive to nanopollutants given their filter-feeding habits [10].

The significant difference between the primary particle size (27 nm) and the hydrodynamic diameter (630 ± 207 nm) is a critical finding that has direct implications for the observed nanotoxicity. The DLS and zeta potential data confirm that ZnONP are not present as individual, discrete particles in the test medium but rather as larger aggregates. This aggregation is a key factor in their environmental fate and bioavailability. The −26.09 mV zeta potential, indicating moderate colloidal stability, suggests that these aggregates are dynamic and can undergo further changes, potentially leading to sedimentation. The ecotoxicological responses observed in *L. fortunei* (such as altered enzyme activities and filtration rate) are therefore likely a response to exposure to these larger agglomerates rather than to the primary individual NP. These findings emphasize the importance of characterizing NP under relevant exposure conditions to accurately interpret their toxic effects and bridge the gap between laboratory results and real-world environmental risks.

Nanozinc has unique properties, such as high reactivity and bioavailability, that bring a complex cascade of effects that become more challenging to elucidate under changing scenarios [44]. In this context, the ZnONP toxicity has been underestimated, as it was previously suggested that non-toxic concentrations of *Limnoperna fortunei* were in the range of 10–50 mg/L [13]; however, according to our results, the LC50-96 h for this species is much lower (11.31 mg/L), and exposure to 25 mg/L caused the mortality of the 100% of the tested organisms (Figure S2). These remarkable differences between results may lie in the intrinsic characteristics of the applied NP. The particles used in this study were dispersed in H_2_O and generated agglomerations when dispersed in the medium. According to Khan et al. [43], ZnONP particles are prone to agglomerate and sediment in heterogeneous matrices, which depend on the temperature (among other variables such as the presence of organic matter). Furthermore, it has been proved that these particles suffer from both increased agglomeration and sedimentation at higher temperatures, thus, affecting the bioavailability for the associated aquatic biota as they become less mobile and easily settle down [4]. Therefore, agglomeration processes could be a key phenomenon that needs further research, especially in a climate-change context of rising temperatures. While studies on another metal-based NP (AgNP) showed that agglomeration in the presence of organic matter and algae may mitigate the toxic effects in freshwater microcrustacean (inhabiting the water column) in terms of life-history traits [30,45], the toxicity mechanisms may be different for benthic and sedentary organisms like the bivalves because it may increase the bioavailability. Another key aspect in terms of exposure conditions consists of the sonication of the particles. In the other study, the ZnONP were sonicated prior to exposure in order to enhance their dispersion in the medium [13], while in this study we did not because: it has been proved that such a procedure has altered (even increased) the toxicity of other metallic NP [46,47], it does not represent the NM reaching the aquatic media, and media with low ionic strength (as freshwater media) guarantee a suitable dispersion.

Tissue damage was generated in the soft tissue of *L. fortunei* by inhibition of enzyme-related activities, which were more evident at 27 °C. To the best of our knowledge, no report is available assessing enzyme tissue damage in mussels exposed to ZnONP, despite the fact that the biomarkers were described to be particularly sensitive in terms of nanopollutants and other stressors (e.g., metals, crude oil, anaerobiosis) [48,49,50,51,52]. Few studies have assessed these biomarkers in freshwater organisms, which make it difficult to discuss these results. Although few studies have been conducted on nanozinc toxicity, Wu and Sokolova [8] exposed *Mytilus edulis* marine mussels to ZnONP and increased temperature, and evidenced an overall blunting of cell responses. Therefore, it could be hypothesized that the temperature may blunt the mechanisms against tissue damage in *L. fortunei*, and thus the intrinsic effects of the nanopollutant may be masked by the increased temperature alone. In the snail *Biomphalaria alexandrina* exposed to high ZnONP concentrations (7, 35 mg/L), the transaminases (ALT, AST) and ALP were found to be drastically increased in hemolymph and soft tissue, and they were related to muscle damage, intestinal and hepatopancreatic injuries, and toxic hepatitis [53]. In the serum of *Cyprinus carpio* fish exposed to nanozinc via intraperitoneal injection (10–20 µg/g), increased activity of the transaminases and ALP were found, and it was related to kidney-related function alterations [54]. Overall, we highlight the much lower concentrations employed in the present study, which may generate differential responses in the organisms for these biomarkers. Furthermore, we consider that waterborne exposure is more environmentally relevant for assessing ecotoxicity than via injection; therefore, this makes it difficult to discuss the obtained findings given the poorly available literature. Lastly, we highlight that a significant decrease in AST in the soft tissue of *L. fortunei* was found at both temperatures analyzed, even at the lowest concentration assayed, which is environmentally relevant (25 µg/L).

Oxidative stress has been widely assessed in terms of nanotoxicity since it is considered one of the main toxicity mechanisms, as ZnONP cellular metabolism generates reactive oxygen species (ROS) and the associated oxidative damage [55]. It must be pointed out that antioxidant-related enzyme activities significantly interacted with the nanozinc concentration and the temperature tested, suggesting that climate change threatens the aquatic ecosystem in complex ways. It has been demonstrated that NP-induced toxicity is related to activation or inhibition of antioxidant enzymes in the case of cytotoxicity triggered by ROS. In this regard, SOD enzyme is the first one to deal with oxyradicals and is responsible for catalyzing the dismutation of the superoxide radical O^2−^ to H_2_O_2_ [55]. Our results showed an increase in enzyme activity after both nanozinc concentrations at 27 °C; however, at 31 °C, its activity remained similar to the control values. The lack of differences in the enzyme activity at the highest temperature could also be explained by a masked effect caused by the high temperature alone toward the nanopollutant, which generated an overall blunt in cell responses [8]. Accordingly, increased SOD activity was observed in the digestive gland of the freshwater mussels *Unio tumidus* exposed for 14 days to ZnONP (concentration expressed as 3.1 µM), which was explained by an enzyme activation due to ROS overproduction [56]. Another study evaluating environmentally relevant concentrations of ZnONP (1 and 10 µg/L) in the marine clam *Ruditapes philippinarum* also reported increased SOD activity in gills and digestive gland [57]. An interesting report carried out by Lai et al. [7] evaluated the interactive effects of ZnONP (concentrations expressed as 0.01 and 3 mg Zn/L) with different ranges of temperature and salinity in the marine mussel *Xenostrobus secures*. The authors found decreased activity of SOD and interaction only with salinity; however, another study that evaluated the ecotoxicity of CeO_2_NP in the marine bivalve *Mytilus galloprovincialis* explained that temperature altered the organisms’ biochemical functions [58]. Key differences in terms of toxicity must be contemplated regarding media with low or high ionic strength (freshwater vs. marine media), making it difficult to assess our findings given the lack of studies on freshwater mussels exposed to nanozinc.

CAT is another important antioxidant enzyme, widely reported, that protects the cell from ROS damage by reducing H_2_O_2_ into water and oxygen; thus, it is crucial to maintain cell homeostasis [59]. Our results showed decreased activity in the soft tissue of *L. fortunei* exposed to the highest ZnONP concentration (250 µg/L) at 31 °C (while no changes were observed at the optimal temperature). As this biomarker showed a significant interaction between the nanozinc exposure, and also for temperature alone, the high temperature could have exacerbated the nanotoxic effects of the particles, as suggested by Morossetti et al. [58] for CeO_2_NP. Accordingly, it was already mentioned that ZnONP could suffer from both agglomeration and sedimentation at higher temperatures [4]. In our study, the TEM analysis showed agglomeration of the ZnONP; thus, a higher temperature could have exacerbated it, leading to higher sedimentation and augmented bioavailability for *L. fortunei* at 31 °C (in comparison with 27 °C). CAT activity inhibition following exposure to nanozinc has been explained by enhanced oxygen free-radical production, and this result is consistent with that reported by Fahmy [53] in hemolymph and soft tissue of the freshwater snail *B. alexandrina* exposed to higher concentrations (7 and 35 mg ZnONP/L). Our results are also consistent with the findings by other authors in muscle of freshwater fish species exposed to nanozinc [60,61].

Remarkably, opposite tendencies were evidenced for GST activity, as it increased at 27 °C but decreased at 31 °C, indicating a clear effect determined by the temperature. Conversely, in the marine mussel *X. securis* exposed to 3 mg ZnONP/L, the opposite behavior was observed (GST activity increased under higher temperatures) [7]; once again, we highlight the difference in terms of the ionic strength of the medium. This enzyme has a key role in the detoxification process, which conjugates reduced glutathione (GSH) with xenobiotics. Therefore, its inhibition (observed at the higher temperature) was explained by a direct action of the metal on the enzyme or indirectly via the ROS production which causes depletion of GSH [57]. The overactivity of GST at the optimal temperature for *L. fortunei* (27 °C) could be due to the need for detoxifying the effects provoked by the metal (Zn ions released by the particles) or the ROS overproduction; however, synergistic effects could have occurred at the highest temperature, bringing additional stress which the enzyme was unable to cope with; therefore, its inhibition happened instead. No effects in terms of lipid peroxidation were observed after exposure to nanozinc at either 27 °C or 31 °C. In other studies conducted on freshwater species exposed to higher ZnONP concentrations, increased LPO levels were observed [53,62]; however, the high tolerance of *L. fortunei*, the exposure time, and the low concentrations selected in this study were not sufficient to cause lipid oxidative damage in the soft tissue of the organisms.

Filtration rate is a crucial characteristic for bivalves that engage in filter feeding and is subject to regulation in response to various environmental factors. In this sense, it has been studied that, despite the high tolerance of *L. fortunei*, temperature is a key factor modifying this behavior [63]. A study conducted on two marine/estuarine mussels exposed to high ZnONP concentrations (2.5–50 mg/L) concluded that changes in the filtration rate are an appropriate indicator for the measurement of nanopollutants [14]. Accordingly, we observed that the control groups for each temperature differed in their filtration rate (it was higher at 31 °C than at 27 °C) as a result of increased physiological processes and metabolism at higher temperature [64]. However, while no differences among the treatments were found at the optimal temperature, a drastic decrease in the filtration rate was observed at 31 °C for both nanozinc concentrations tested. According to Quevedo et al. [65], macroinvertebrate assemblages are sensitive to thermal changes in a context of climate change (even when temperature does not exceed 3 °C). This could be a crucial threat for filter-feeding species with ultimate implications for their body storage, as has been proven for *L. fortunei* exposed to Cu and a wide range of temperatures [22]. The low filtration rate could be explained by a synergistic effect provoked by the nanozinc exposure and the higher temperature (not evidenced at 27 °C). In the case of a harmful environment, the bivalves are capable of minimizing their water contact by reducing the valve opening period and narrowing valve gap amplitude, resulting in a reduced filtration rate [7]. Studies on mollusks exposed to other metal-based NP (TiO_2_NP, CuONP) also reported a decreased filtration rate, which was related to a reduction in energy consumption [66].

It has also been documented that *L. fortunei* is able to selectively ingest and retain particles spanning a broad size range (approximately 1–1000 µm), while smaller particles (≤1 µm) are typically aggregated and expelled as pseudofeces [67,68]. In this sense, exposure to TiO_2_NP has been reported to stimulate the secretion of mucus filaments in *L. fortunei* and *M. edulis*, suggesting an adaptive mechanism to entrap the particles, thus limiting their assimilation and mitigating the toxic effects [69,70]. Furthermore, another behavioral mechanism described in bivalves to reduce the pollutant uptake and maintain the homeostasis under stress conditions is the valve closure [67]. Therefore, these defense mechanisms could have prevented the filtration rate alteration in *L. fortunei* under the optimal temperature; however, they were not sufficient to prevent deleterious effects at 31 °C, as the joint effect with the nanopollutant could have exceeded the bivalves’ tolerance to stress. Lastly, it has been explained that particle accumulation may have an effect on ciliary beating and muscular changes in gills, which are physiologically controlled by the nervous system [71]; in this regard, further studies are needed on ZnONP and their neurological effects on mollusks.

## 5. Conclusions

Overall, we highlight the ecotoxicological implications of nanozinc in a climate-change context, even at environmentally relevant concentrations (such as the lowest one tested in this study). Nanozinc was proven to cause tissue damage, disrupt the redox homeostasis, and alter the filter-feeding habits in the golden mussel *L. fortunei*; these effects may become more complex in a changing world of rising temperatures. We consider that bivalves are key organisms for evaluating exposure to nanopollutants, as their sensitivity and filter-feeding habits are valuable for obtaining early responses. With the freshwater habitats being poorly represented among the available literature, complex mechanisms for emerging contaminants like the NM are needed to be elucidated in the near future.

## Figures and Tables

**Table 1:** Summary of the 2-way ANOVA applied to evaluate the effects of nanozinc concentration, temperature, and their interaction (Conc × Temp) on biomarker responses of *L. fortunei*. Significant effects are in **bold** at *p* < 0.05.

Biomarker	Factor	df	F Value	*p* Value
ALT	Concentration	2	0.89	0.4246
	Temperature	1	0.81	0.3786
	Conc × Temp	2	0.16	0.8555
AST	Concentration	2	8.5	**0.0018**
	Temperature	1	2.27	0.1462
	Conc × Temp	2	1.41	0.2655
ALP	Concentration	2	2.28	0.1242
	Temperature	1	9.95	**0.0043**
	Conc × Temp	2	1.27	0.3001
SOD	Concentration	2	1.41	0.2663
	Temperature	1	0.64	0.4325
	Conc × Temp	2	8.11	**0.0026**
CAT	Concentration	2	1.69	0.2091
	Temperature	1	12.41	**0.002**
	Conc × Temp	2	3.86	**0.0373**
GST	Concentration	2	1.79	0.1917
	Temperature	1	2.21	0.152
	Conc × Temp	2	8.96	**0.0015**
LPO	Concentration	2	1.9	0.1713
	Temperature	1	1.26	0.2727
	Conc × Temp	2	0.26	0.7705

**Table 2:** Summary of the GLM applied to evaluate the effects of nanozinc concentrations and temperature after 3, 6 and 12 h on filtration rate of *L. fortunei*. Significant effects are in **bold** at *p* < 0.05.

Contrast			Ratio	SE	*t* Ratio	*p* Value
3 h						
Control	27 °C/0.025 µg/L	27 °C	1.177	0.445	0.431	0.9979
Control	27 °C/0.250 µg/L	27 °C	1.511	0.665	0.938	0.9337
Control	27 °C/control	31 °C	0.766	0.242	−0.843	0.9570
Control	27 °C/0.025 µg/L	31 °C	1.074	0.388	0.197	1.0000
Control	27 °C/0.250 µg/L	31 °C	0.906	0.304	−0.295	0.9997
0.025 µg/L	27 °C/0.250 µg/L	27 °C	1.284	0.595	0.539	0.9941
0.025 µg/L	31 °C/control	31 °C	0.650	0.226	−1.236	0.8160
0.025 µg/L	31 °C/0.025 µg/L	31 °C	0.912	0.355	−0.236	0.9999
0.025 µg/L	31 °C/0.250 µg/L	31 °C	0.769	0.281	−0.717	0.9785
0.250 µg/L	31 °C/control	31 °C	0.507	0.211	−1.631	0.5845
0.250 µg/L	31 °C/0.025 µg/L	31 °C	0.711	0.321	−0.756	0.9729
0.250 µg/L	31 °C/0.250 µg/L	31 °C	0.599	0.258	−1.188	0.8395
Control	31 °C/0.025 µg/L	31 °C	1.402	0.459	1.033	0.9035
Control	31 °C/0.250 µg/L	31 °C	1.183	0.353	0.562	0.0028
0.025 µg/L	31 °C/0.250 µg/L	31 °C	0.844	0.292	−0.491	0.9962
6 h						
Control	27 °C/0.025 µg/L	27 °C	1.170	0.563	0.327	0.9995
Control	27 °C/0.250 µg/L	27 °C	1.123	0.532	0.245	0.9999
Control	27 °C/control	31 °C	0.317	0.107	−3.404	**0.0196**
Control	27 °C/0.025 µg/L	31 °C	1.279	0.646	0.488	0.9963
Control	27 °C/0.250 µg/L	31 °C	1.177	0.567	0.339	0.9994
0.025 µg/L	27 °C/0.250 µg/L	27 °C	0.959	0.488	−0.081	1.0000
0.025 µg/L	31 °C/control	31 °C	0.271	0.1044	−3.392	**0.0202**
0.025 µg/L	31 °C/0.025 µg/L	31 °C	1.093	0.588	0.165	1.0000
0.025 µg/L	31 °C/0.250 µg/L	31 °C	1.006	0.519	0.011	1.0000
0.250 µg/L	31 °C/control	31 °C	0.282	0.107	−3.352	**0.0223**
0.250 µg/L	31 °C/0.025 µg/L	31 °C	1.139	0.606	0.245	0.9999
0.250 µg/L	31 °C/0.250 µg/L	31 °C	1.048	0.535	0.093	1.0000
Control	31 °C/0.025 µg/L	31 °C	4.038	1.670	3.366	**0.0215**
Control	31 °C/0.250 µg/L	31 °C	3.712	1.430	3.400	**0.0198**
0.025 µg/L	31 °C/0.250 µg/L	31 °C	0.920	0.496	−0.154	1.0000
12 h						
Control	27 °C/0.025 µg/L	27 °C	0.998	0.239	−0.007	1.0000
Control	27 °C/0.250 µg/L	27 °C	2.042	0.736	1.981	0.3736
Control	27 °C/control	31 °C	1.035	0.251	0.143	1.0000
Control	27 °C/0.025 µg/L	31 °C	1.407	0.397	1.211	0.8284
Control	27 °C/0.250 µg/L	31 °C	1.353	0.373	1.095	0.8798
0.025 µg/L	27 °C/0.250 µg/L	27 °C	2.045	0.736	1.987	0.3704
0.025 µg/L	31 °C/control	31 °C	1.037	0.251	1.150	1.0000
0.025 µg/L	31 °C/0.025 µg/L	31 °C	1.409	0.397	1.218	0.8252
0.025 µg/L	31 °C/0.250 µg/L	31 °C	1.355	0.374	1.102	0.8771
0.250 µg/L	31 °C/control	31 °C	0.507	0.184	−1.867	0.4386
0.250 µg/L	31 °C/0.025 µg/L	31 °C	0.689	0.268	−0.959	0.9277
0.250 µg/L	31 °C/0.250 µg/L	31 °C	0.663	0.255	−1.071	0.8893
Control	31 °C/0.025 µg/L	31 °C	1.359	0.388	1.075	0.8879
Control	31 °C/0.250 µg/L	31 °C	1.307	0.365	0.958	0.9280
0.025 µg/L	31 °C/0.250 µg/L	31 °C	0.962	0.301	−0.125	1.0000

## Data Availability

The raw data supporting the conclusions of this article will be made available by the authors upon request.

## Supplementary Materials

The following supporting information can be downloaded at: https://www.mdpi.com/article/10.3390/ani15182734/s1, Figure S1: Map showing the collection area of *L. fortunei* in Santa Fe River, Argentina (31°38′34.90″ S; 60°41′6.22″ W), Figure S2: Lethal concentration 50 (LC50, mg ZONP/L), 95% confidence intervals, and dose–response curve in *L. fortunei*.
