# Supplementary material for: Nanozinc Ecotoxicity in the Freshwater Invasive Bivalve Limnoperna fortunei Under a Climate Change Scenario
Source: Animals (Basel). 2025 Sep 19;15(18):2734. doi: 10.3390/ani15182734 (PMC12466390; doi:10.3390/ani15182734)
Supplement: Supplementary file 1 [file animals-15-02734-s001.zip › animals-3869687-supplementary.pdf]

SUPPORTING INFORMATION

Nanozinc Ecotoxicity in the Freshwater Invasive Bivalve *Limnoperna fortunei* Under a Climate Change Scenario

Analía Ale [1,2,*], Victoria S. Andrade [3], Florencia M. Rojas Molina [3,4], Luciana Montalto [3,4], Lucía M. Odetti [1,4], Pablo E. Antezana [5], Martín F. Desimone [6] and María Fernanda Simoniello [1]

1 Cátedra de Toxicología, Farmacología y Bioquímica Legal, Facultad de Bioquímica y Ciencias Biológicas, Universidad Nacional del Litoral (FBCB-UNL), Santa Fe S3000, Argentina; luodetti@gmail.com (L.M.O.); fersimoniello@yahoo.com.ar (M.F.S.)

2 Consejo Nacional de Investigaciones Científicas y Técnicas (CONICET), Buenos Aires C142F, Argentina

3 Instituto Nacional de Limnología, Consejo Nacional de Investigaciones Científicas y Técnicas (CONICET), Universidad Nacional del Litoral (INALI-CONICET-UNL), Santa Fe S3000, Argentina; vandrade@inali.unl.edu.ar (V.S.A.); florojasm@yahoo.com.ar (F.M.R.M.); lmontalto@inali.unl.edu.ar (L.M.)

4 Facultad de Humanidades y Ciencias, Universidad Nacional del Litoral (FHUC-UNL), Santa Fe S3000, Argentina

5 Instituto de Bioquímica y Medicina Molecular, Facultad de Farmacia y Bioquímica, Universidad de Buenos Aires (FFyB-UBA), Consejo Nacional de Investigaciones Científicas y Técnicas (CONICET), Buenos Aires C1113, Argentina; pablo.e.antezana@gmail.com

6 Instituto de Química y Metabolismo del Fármaco, Facultad de Farmacia y Bioquímica, Universidad de Buenos Aires (IQUIMEFA-FFyB-UBA), Consejo Nacional de Investigaciones Científicas y Técnicas (CONICET), Buenos Aires C1113, Argentina; desimone@ffyb.uba.ar

* Correspondence: aale@fbcb.unl.edu.ar

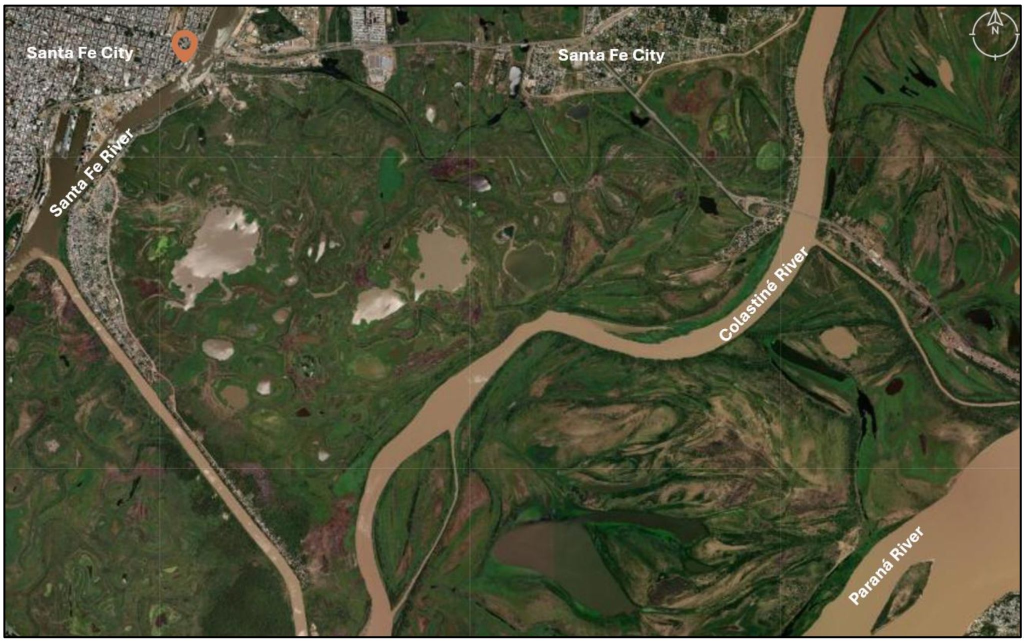

Figure S1. Map showing the collection area of *L. fortunei* in Santa Fe River, Argentina (31°38'34.90" S; 60°41'6.22" W).

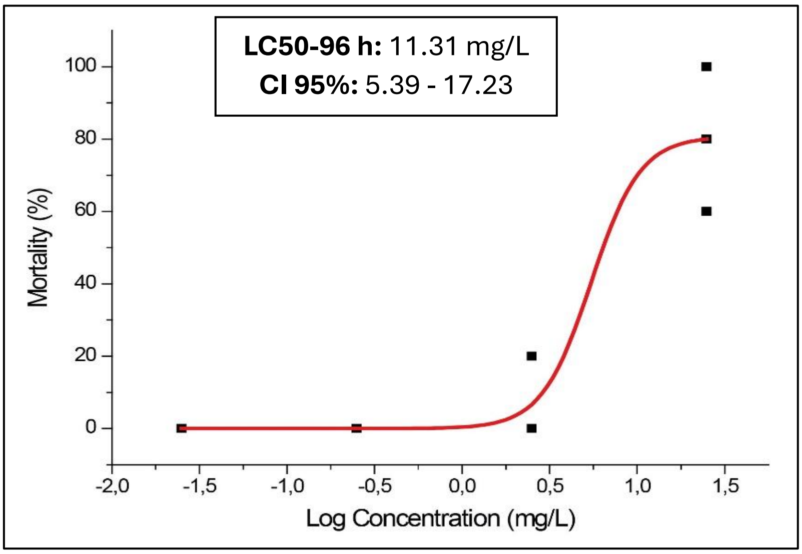

Figure S2. Lethal concentration 50 (LC50, mg ZnONP/L), 95% confidence intervals (CI), and dose-response curve in *L. fortunei.*
